# Spatial variability of soil nutrients in seasonal rivers: A case study from the Guo River Basin, China

**DOI:** 10.1371/journal.pone.0248655

**Published:** 2021-03-16

**Authors:** Cangyu Li, Xinhui Wang, Mingzhou Qin

**Affiliations:** 1 The College of Environment and Planning, Henan University, Kaifeng, China; 2 College of Plant Protection, Henan Agricultural University, Zhengzhou, China; Soil and Water Resources Institute ELGO-DIMITRA, GREECE

## Abstract

Agricultural non-point source pollution refers that substance such as nitrogen and phosphorus cause water environment pollution through surface runoff and underground leakage in agricultural production activities. Water environment pollution related to agricultural non-point source pollution in the Huaihe River Basin is becoming more and more prominent. Therefore, it is necessary to analyze the characteristic of soil nutrient in cultivated land and explore the spatial variation and influencing factors of soil nutrients at the watershed scale. A total of 239 topsoil samples were collected from the Guo river basin, and the related factors of soil organic matter (SOM), total carbon (TC), total nitrogen (TN), total phosphorous (TP), total potassium (TK) and potential of hydrogen (PH) were studied by using descriptive statistics and geostatistical methods. The results showed that TK and PH were weak variation, while SOM, TC, TN and TP were medium variation. Soil pH, TP, TK, TC and SOM had moderate spatial variability, which was caused by both random factors and structural factors such as soil texture, soil type, fertilization and local ecological restoration management. Soil TN showed a strong spatial correlation, mainly due to soil texture and soil type. If the recommended fertilization amount is still given based on the average value of soil nutrients ignoring the spatial heterogeneity, it will not only affect crop production efficiency and fertilizer utilization, but may also cause greater environmental pollution. This study can provide a theoretical basis for the management of agro-ecological environments throughout the basin area.

## Introduction

Soil plays a crucial role in biogeochemical cycles as an important source and sink of nutrients. Soil properties exhibit a complex degree of variability in both space and time [1]. Such variability is both continuous across the landscape and scale-dependant because soil properties are the outcome of the combined interaction of biological, chemical and physical processes acting at multiple scales. The study of the spatial variability of soil properties is to quantify the complexity of the spatial variability. Carrying out this research can not only improve or innovate the soil classification system, but also improve the accuracy of soil survey, mapping and field trials.

In recent years, the study of the spatial distribution of soil nutrients has always been a hot issue. Due to the limitation of the number of sampling points, it is necessary to check the non-sampling points. The past research methods for and spatial variability of soil nutrients include traditional statistics method, the neural network method, the geographic information technology method, high-precision surface modeling method and geostatistical method. These interpolation methods have their own advantages and disadvantages. Traditional statistics can only reflect the global characteristics of soil nutrients, but cannot represent local characteristics, and it is difficult to reflect the spatial distribution pattern of soil nutrients accurately. The neural network method can improve the accuracy of the spatial variation at a small scale, but there are still some urgent problems need to be solved. For example, the BP (Back Propagation) neural network [2] is easy to fall into a local minimum. As for the RBF (Radial Basis Function Neural Network) neural network [3], the calculation of weights from the hidden layer to the output layer seriously affects the accuracy of its spatial interpolation. The geographic information technology method adds environmental information such as terrain, climate, and hydrology to the study of the spatial variability of soil nutrients, which improves the accuracy at a certain extent. However, the environmental information at a large scale must rely on 3S technology, and spatial data has uncertainties, DEM (Digital Elevation Model) also has scale effects [4]. The high-precision surface modeling method based on surface theory has been introduced into the studies for spatial variability of soil nutrient in recent years. However, there are two major difficulties in the application process: high computational complexity and high storage capacity. Amounts of studies report that geostatistics is considered to be one of the most effective methods to study the variation and spatial distribution characteristics of soil nutrients [5]. Geostatistics is a technology for estimating the soil property values in nonsampled areas or areas with sparse samplings [6]. These non-sampled areas can vary in space (in one, two or three dimensions) from the sampled data. Geostatistics provides a set of statistical tools for a description of spatial patterns, quantitative modeling of spatial continuity, spatial prediction, and uncertainty assessment [7]. Geostatistical techniques incorporating spatial information into prediction can improve estimation and enhance map quality. Although the majority of standard geostatistical techniques have been applied in small-scale areas including describing the spatial distribution of soil [8] and analyzing spatial variability of soil properties [9], few applications in large-scale were estimated [10].

Geostatistical methods with spatial interpolation can predict spatial distribution. The studies for spatial variability of soil nutrients using geostatistical methods mainly contain the semivariogram and Kriging interpolation. The semivariogram model for fitting soil nutrients can better reflect the spatial variation structure of soil nutrients and directly affect the accuracy of Kriging interpolation. Kriging was originally developed in geostatistics by Danie Krige, a South African mining engineer. It is an interpolation method derived from the theory of regional variable, which is based on a statistical model of the statistical relationship between measured locations and reflects spatial correlation of surface changes [11]. The Kriging method relies on the graph of variance to represent the spatial variation and minimizes the prediction error. Kriging is more suitable for data obtained from experimental areas with larger regions than those used in lower-order polynomial regression models [12]. The Kriging interpolation method based on geostatistics is currently the most widely used method in soil fertility evaluation. In recent years, many scholars use semivariogram to fit the spatial variability model of soil nutrients, and use Kriging interpolation to quantitatively predict the spatial distribution of soil nutrients. For example, Wang (2016) [13] studied the spatial variability of SOM, pH, TN, available nitrogen, available phosphorus, and available potassium in Zhengpugang New Area, Anhui Province using geographic information systems method combined with geostatistics. Bai (2016) [14] studied the spatial distribution characteristics of nitrogen in samples of different soil types in the Yinghe River Basin and their relationships with various influencing factors, such as soil type, land use type, nitrogen fertilizer application, rainfall, groundwater depth, etc. Wang (2005) [15] used a combination of traditional statistics and geostatistics to analyze the spatial variability of soil nutrients and pH in the cultivated layer of vegetable fields at Taihu Lake. Wang et al (2003) [16–18] used geostatistics to quantitatively study the spatial variability of soil nutrients in the Danangou watershed of the Loess Plateau, and analyze the spatial distribution of SOM, TN, TP in the study area using Kriging interpolation.

Kriging interpolation methods mainly include simple Kriging, ordinary Kriging, universal Kriging, indicator Kriging, disjunctive Kriging, co-Kriging, etc. The simple Kriging method requires that the mean value of soil properties in the study area is constant and known, which conforms to the stability assumption. However, the average value is generally difficult to obtain. The ordinary Kriging method requires the average value of the soil properties of the study area to be constant and unknown, which is the most robust and most commonly used method. The universal Kriging method requires the average value to be variable and unknown. However, it needs to predict the residual variance function, which is also the main reason that restricts its wide application. The disjunctive Kriging method can predict the probability of a given threshold that both exceed or lower the true value of unknown points or blocks. This method is widely used in the field of environmental risk assessment. The co-kriging method is mainly used to use easily available soil properties to predict another soil that is highly correlated and difficult to obtain Property [19]. Overall, Ordinary Krging method is currently the most widely used method in the research of soil nutrient interpolation. Many scholars have used this method to study the spatial distribution characteristics of soil nutrients [20,21].

Seasonal rivers refers are dry during a certain season of the year or for a long period of time. The Kaifeng section of the Guo River has the characteristics of a seasonal river and is a man-made seasonal river. It is a new type that has emerged under the influence of human activities in modern times. In addition, the Guo River Basin is also one of the main food production areas. Proving its nutrient distribution has become an important requirement for increasing food production, reducing food production costs, and planning agricultural production areas. It is important for strengthening agricultural non-point source pollution control (especially with water environment pollution related to agricultural non-point source pollution and sustainable land use also play a significant role, It can also provide reference value for soil nutrient management in other similar regions. Although scholars have done some related research works on the spatial variability of soil properties at the small watershed scale [22–25], there are still some application limitations, such as the excessive workload of measured data, the instability of the soil properties in complex areas and Seamless connection of multi-source data. The soil nutrients in this study are relatively easy to obtain and the mean value is unknown, which is more suitable for interpolation analysis using ordinary Kriging method. The spatial variability of soil nutrients in different regions is quite different. Although many scholars have used geostatistical methods to explore the spatial distribution of soil nutrients in other regions, there is no research on the spatial variability in the Guo River Basin.

In this study, we used geostatistical methods to study the spatial distribution of soil nutrients in seasonal river basins based on field investigation and sampling analysis. We evaluated the variation characteristics and delineated the distribution of soil chemical properties such as soil organic matter (SOM), total carbon (TC), total nitrogen (TN), total phosphorous (TP), total potassium (TK) and pH using the Kriging interpolation. The purposes of this study: 1) to explore the distribution of soil nutrients in the Guo River Basin; 2) to analyze the characteristics and influencing factors of spatial variability in the Guo River Basin.

## Materials and methods

### Site description

**Fig 1:**
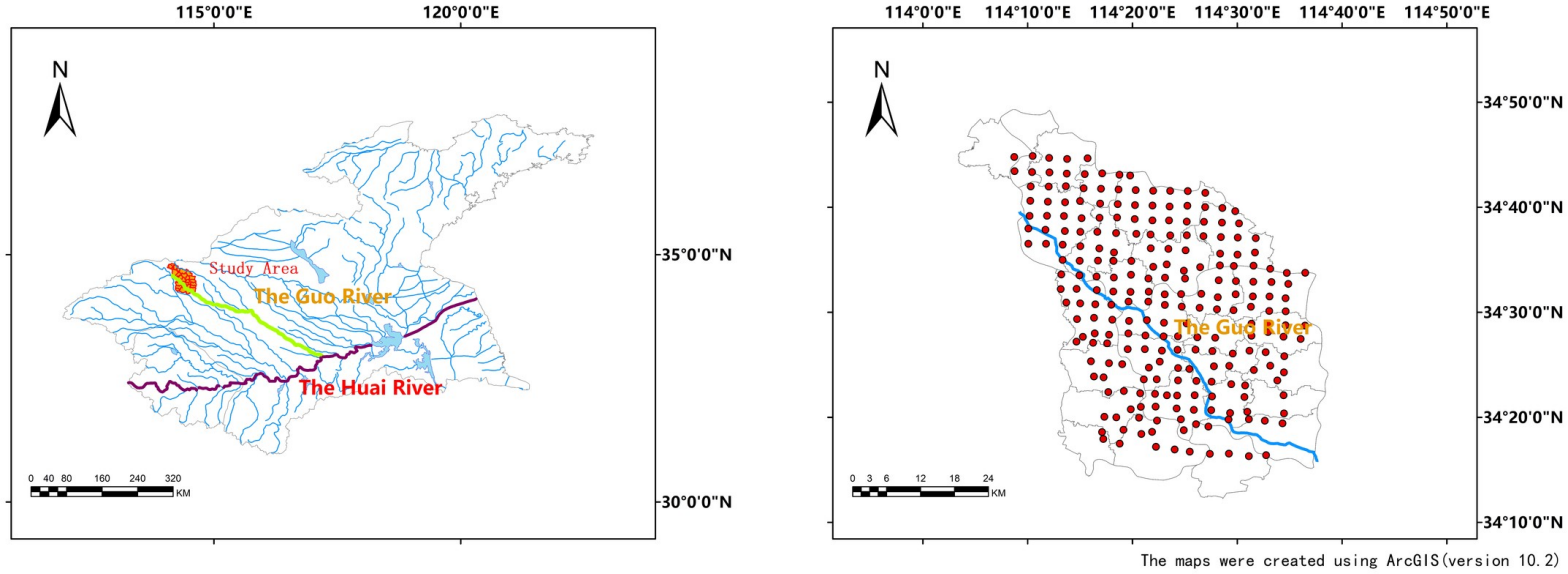
Study area and sampling sites in the Guo River basin.

The Huaihe River Basin is one of the most developed industrial and agricultural regions in China. While developing the economy in this region, it also brings tremendous pressure to the ecological environment of the basin, especially the water environmental pollution related to agricultural non-point source pollution such as nitrogen and phosphorus. The problem is more prominent. The Guo River Basin belongs to the backbone drainage channel of the Huai River Basin across the Henan and Anhui provinces. It originates from Xukou Town, Xijiangzhai Township, Xiangfu District, Kaifeng City, Henan Province, and flows through Kaifeng County, Kaifeng, Tongxu (Fig 1).

At Kaifeng, the Guo River has a length of 72.22 km, with a drainage area of 105,200 hm^2^ and a cultivated area of 73,600 hm^2^. The terrain is relatively flat, with an undeveloped water system, which is not conducive to the formation of surface runoff. The annual variation of river runoff mainly depends on the seasonal variation of precipitation, and the rainfall in the Guo River Basin is concentrated from July-September, accounting for 58% of the total rainfall, making the Guo River a seasonal river. Surface runoff mainly occurs from July to September, in the flood season. The annual distribution of the measured runoff in Xuanwu is extremely uneven; the average annual maximum monthly runoff is 44.73 million m^3^ (August), while the minimum monthly runoff is only 2.22 million m^3^ (April). In the first half of May, with gradually increasing precipitation, river runoff starts to increase, while after October, it decreases rapidly. The interannual variation of the measured runoff is also highly significant. It is one of the areas with the most frequent agricultural activities. In recent years, according to the statistics of the scalar amount of chemical fertilizers in Kaifeng, the total amount of chemical fertilizers applied in Kaifeng in 2018 reached 236,234t, of which the amount of nitrogen fertilizer was 98,754t and the amount of phosphate fertilizer was 46,881t. The application rate of potash fertilizer is 25,705t, and the application rate of compound fertilizer is 64,894t; the application rate of chemical fertilizer per unit area is 344kg/hm^2^, which is higher than 328.5kg/hm^2^ of chemical fertilizer per unit of crops in my country, and far higher than the recognized safe level of 225kg/hm^2^ in developed countries. Among various land use methods, the planting area of agricultural products is expanding year by year. Due to the large amount of soil fertilizer input, and most of the arable land in the watershed adopts winter wheat and summer maize cultivation methods, the multiple cropping index is high, and frequent irrigation, etc., not only cause a large amount of nitrogen and phosphorus to be lost with the water into the water body of the Guo The eutrophication of water bodies also leads to the accumulation of nitrate in agricultural products, potentially threatening the health of humans and animals. Therefore, it is necessary to analyze and study the soil nutrient status of cultivated land in this area.

### Soil sampling and chemical analysis

Based on the data of the soil type map and on the topography and geomorphology of Henan Province, as well as on the second soil survey data and soil fertility monitoring data, the soil is sampled after the local crops are harvested considering that the soil type and distribution characteristics to avoid over-fertilization.

In August 2018, adopting the mesh uniform distribution mode and using a sampling grid of 2.5 x 2.5 km, a total of 239 topsoil samples was collected along the Guo River Basin at Kaifeng section (34°11′47″N-34°48′40″N, 114°4′13″E-114°39′2″E) (Fig 1). The figures were all created using ArcGIS (version 10.2) software. Each sample point was sampled five times, collecting the surface layer (0–20 cm) of the ploughed soil and taking an amount of 1 kg. For each sample point, we recorded latitude, longitude, date, sampling depth, soil type, crop type, crop yield level, fertilization management status, planting institution, topography, irrigation condition and farmland facility. Soil samples were air-dried, ground and sieved with 0.149, 0.25 and 2 mm meshes for chemical analysis. SOM was determined using the Walkley and Black method [26]. TN was ananlyzed with the Kjeldahl procedure [27]. TP was measured following the molybdenum blue colorimetric method. TK was determined using flamephotometer. The potentiometric method was used to determine pH.

### Classical statistics and geostatistical analysis

SAS (Statistical Analysis System) software was utilized for a correlation analysis and variability in the soil properties was analyzed using the minimum, maximum, mean, median, standard deviation, and coefficient of variation. Before the geostatistical analysis, spatial distances of soil sample locations were determined. These were then entered into Arcview software, and the latitudes and longitudes were transformed to Universal Transverse Mercator (UTM) distances. Based on the sampling and index test errors, the Grubbs method is used to identify and process the most severe outliers, and the abnormal values are eliminated [28]. The subsequent correlation calculations use the data processed by the outliers. Normal distribution is the basis for Kriging spatial analysis. SPSS 19.0 software with the non-parametric test (α = 0.05) was used to determine whether it conforms to normal distribution. The calculation of the semivariogram function and the fitting of the theoretical model were performed using the geostatistical software GIS 10.0 [29] with spatial autocorrelation analysis.

In geostatistics, the spatial variability of a variable can be characterized by a semivariogram function and the calculation of its function can be expressed based on the following equation [30]:

$$\text{r}(\text{h})=\frac{1}{2N(h)}\sum_{i\;=\;1}^{N(h)}{\left[Z(x_{i})-Z(x_{i}+h)\right]}^{2}$$ (1)

where r(h) is the semivariogram function, h is the spatial distance of the sample points, called the step size, N(h) is the number of samples with the separation distance h, Z(xi) and Z(xi +h) are the measured value of the regionalized variable Z(xi) at the spatial positions xi and xi + h, respectively. If h is the abscissa, the function graph drawn with r(h) as the ordinate is called the semivariogram function graph. The fitting of the r(h) coordinate value can be used to obtain the corresponding theoretical model and the model parameters. The characteristics of spatial variability can be obtained by analyzing the model parameters. The fitting of the semivariogram model of the variable generally requires that the coefficient of determination (R^2^) of the fitted model is large and that the residual (RSS) is small. The theoretical models fitted by the commonly used semivariogram function are Spherical, Exponential and Gaussian models. The models are as below [31]:

$$\text{r}(h)\;=\;\{\begin{array}{c} 0,h=0\\ C_{0}+C(\frac{3h}{2a}-\frac{h^{3}}{2a^{3}}),0\leq h\leq a\\ C_{0}+C,h>a \end{array}$$ (2)

where C_0_ is the nugget constant, C is the partial base station, C_0_ + C is the base value, and a is the theoretical range. The Spherical model shows that the spatial autocorrelation gradually decreases with the increase of distance and becomes zero after a certain distance is exceed.

$$\text{r}(h)\;=\;\{\begin{array}{c} 0,h=0\\ C_{0}+C(1-e^{\frac{-h}{a}}),h>0 \end{array}$$ (3)

The theoretical range corresponding to the Exponential model is 3a. The spatial autocorrelation of the model decreases exponentially with the increase of distance, and the autocorrelation will only disappear completely at infinity

$$\text{r}(h)\;=\;\{\begin{array}{c} 0,h=0\\ C_{0}+C(1-e^{\frac{-h^{2}}{a^{2}}}),h>0 \end{array}$$ (4)

The theoretical range corresponding to the Gaussian model is 3a. The model shows that the spatial autocorrelation firstly increases as the distance increases, and then gradually decreases, and becomes zero after a certain distance is exceeded.

The nugget value of the theoretical model is the variation caused by the experimental error and less than the actual sampling scale, indicating the spatial heterogeneity of the random part [32]. A large nugget value indicates that some process on the smaller scale cannot be ignored. The base value are usually expressed the total variation within the system. The ratio of the nugget value to the abutment value can indicate the degree of correlation of the system variables. If the ratio is < 25%, the system has a strong spatial correlation; if the ratio is between 25 and 75%, the system is described. A moderate spatial correlation (> 75%) indicates that the system correlation is weak [33]. The range is the distance corresponding to the variogram reaching the abutment value, which indicates the spatial autocorrelation range of the soil nutrients. Ordinary kriging spatial interpolation of soil nutrients and pH in this study area was carried out using the ArcGIS 10.2 platform.

## Results

### Descriptive statistical characteristics and trend analysis of soil nutrients and pH value

**Fig 2:**
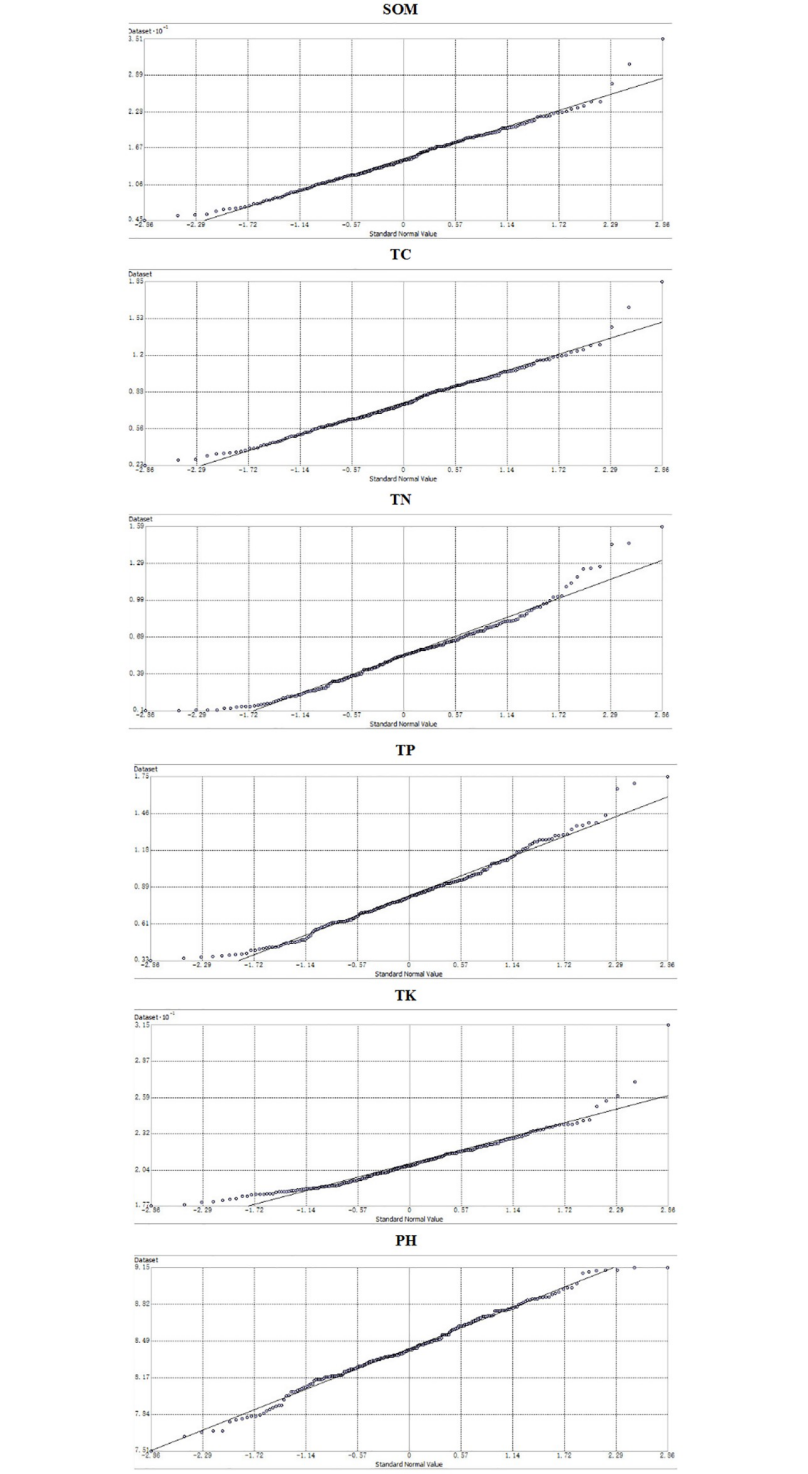
Normal distribution map of soil nutrients.

Table 1 shows the classical statistical characteristics of soil nutrient. The results show that the average values of SOM, TC, TN, TP, and TK were 14.85, 0.78, 0.54, 0.83, and 20.90 g/kg, respectively. According to the nutrient classification standard of the second national soil survey, the content of TK and TP reached the second high level standard, the SOM content was at the middle to low level of the fourth grade, and the TN content was at the low level of the fifth grade. The coefficient of variation (CV) reflects the degree of dispersion of the data, with a value lower than 10% indicating a weak variation, a value between 10 and 90% indicating a moderate variation, and a value above 90% indicating a strong variation [34]. Based on Table 1, the spatial variability of TK and pH was relatively low, with 8.7 and 3.7%, respectively. The parameters SOM, TC, TN, and TP showed moderate variation. Overall, the soil parameters followed the order TN > TP > TC > SOM > TK > pH. The spatial variation of SOM, TN, and TP in the seasonal watershed of the Kaifeng section was most likely influenced by human activities such as farming or land use. The log-transformed data of the five soil nutrients followed a normal distribution (Fig 2). At the same time, the non-parametric test was carried out by the K-S verification method (Table 2), which indicated that the soil nutrients were consistent with the positive distribution.

**Table 1:** Descriptive statistics of soil nutrients.

	Sample No	(g/kg)Min	(g/kg)Max	(g/kg)Mean	(g/kg) SD	(g/kg) D(X)	Skewness	Kurtosis	CV%
**SOM**	239	4.48	35.07	14.85	4.71	22.02	0.47	0.99	**31.71**
**TC**	239	0.23	1.85	0.78	0.25	0.06	0.49	1.05	**32.05**
**TN**	239	0.10	1.59	0.54	0.27	0.07	0.75	1.16	**50.00**
**TP**	239	0.33	1.75	0.83	0.27	0.07	0.51	0.41	**32.53**
**TK**	239	17.66	31.47	20.90	1.83	3.33	1.21	4.17	**8.70**
**PH**	239	7.51	9.15	8.43	0.32	0.10	-0.13	-0.07	**3.70**

**Table 2:** Kolmogorov-Smirnov test.

		SOM	TC	TN	TP	TK	PH
**N**		239.00	239.00	239.00	239.00	239.00	**239.00**
**GDP** ^a^^,^^b^	Mean	14.85	0.78	0.54	0.83	20.90	**8.43**
	SD	4.71	0.25	0.27	0.27	1.83	**0.32**
**Most extreme difference**	ABS	0.04	0.04	0.05	0.05	0.06	**0.04**
	positive	0.04	0.04	0.05	0.05	0.05	**0.03**
	negative	-0.02	-0.02	-0.05	-0.03	-0.06	**-0.04**
**Kolmogorov-Smirnov Z**		0.63	0.68	0.74	0.75	0.87	**0.65**
**Asymptotically significant**		0.82	0.75	0.64	0.63	0.44	**0.80**

GDP. Gaussian distribution parameter,

^a^ The test distribution is a Gaussian distribution,

^b^ Calculated based on the data.

### Trend analysis of soil nutrients and pH in the study area for Kriging

**Fig 3:**
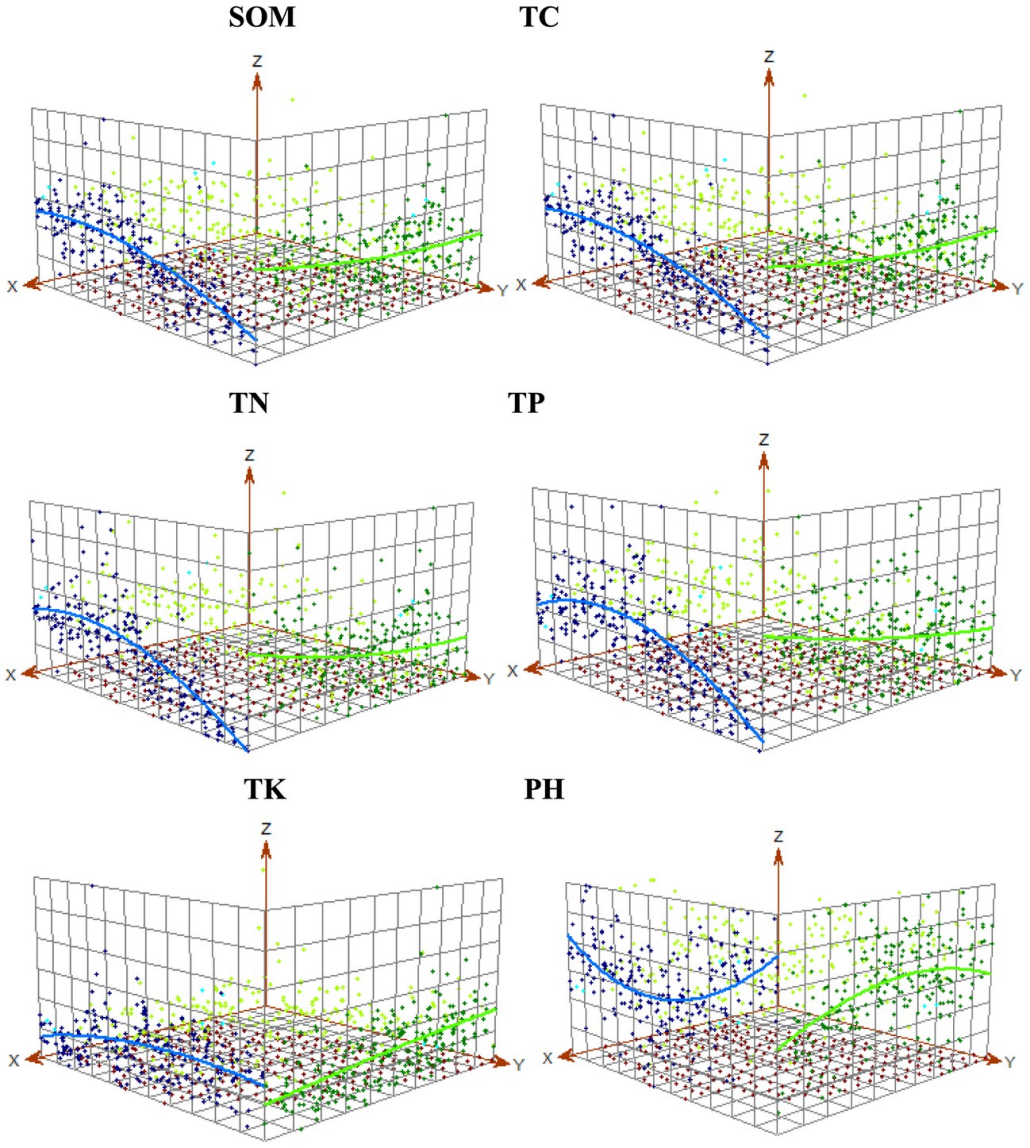
Trend analysis of soil nutrient contents.

To reduce the impacts of total trends on the local semi-variant analysis process and more accurately simulate short-range random variation, trend surface analysis was performed in order to obtain soil nutrient trends. Based on the Kriging interpolation, the trend analyses of soil nutrient content and pH in the study area were carried out in Fig 3. Each point represents the attribute value of a sample. These points were then projected onto the EW SN orthogonal plane, and the best fit line was made through the projection point. The X axis represents the east direction, the Y axis represents the true north direction and the Z axis indicates the magnitude of the measured value of each sample. The green curve indicates the change of the trend effect of the east-west trend and the blue curve the change of the trend effect of the south-north direction. When simulating trends that exist in a particular direction, and if the line is straight, there is no global trend. SOM and TC showed a downward trend from east to west (Fig 3). The contents of TN and TP followed a U-shaped distribution from south to north, with a decrease from east to west (Fig 3). The TK content slightly increased from south to north and from east to west, while pH showed a U-shaped trend from south to north and an inverted U-shaped trend from east to west (Fig 3).

### Spatial distribution of soil nutrients and pH

The semi-variogram can indicate the spatial variability of soil properties. Table 3 shows the corresponding parameters obtained from the theoretical model of each element variogram. The parameter C_0_ represents the block gold variance (half variance when the interval is 0), which is caused by the experimental error and the random factors such as fertilization, crop, and management level on the sampling scale. (C + C_0_) is the structural variance and caused by non-human structural factors such as climate, vegetation type, soil parent material and topography. C_0_/(C + C_0_) indicates the degree of spatial variability of soil nutrients and a high level indicates that the degree of spatial variability caused by random parts is large and caused by structural factors. When C_0_/(C+C_0_) < 25%, the variable has a strong spatial correlation and the spatial variability is mainly caused by endogenous variation (random factors). If C_0_/(C+C_0_) is between 25% and 75%, the variable has a moderate spatial correlation and the spatial variability may be controlled by internal and external factors. When C_0_/(C+C_0_) >75%, the spatial correlation is relatively weak and the spatial variability is mainly caused by exogenous variation (non-human structural factors) [35]. In Table 3, the R^2^ values of soil pH, TN, TP, SOM and TC were all greater than 0.9, indicating that the spatial distribution of them was more stable, which may be caused by human factors.

**Table 3:** Parameters and models of fitted semivariograms of soil nutrient concentrations.

	Nugget Co	Partial sill C Sill(Co+C)	Range (km)	Theoretic model	R^2^	Nugget to sill ratio % (%) /Co/(Co+C)
**PH**	0.0464	0.1138	24	Spherical	0.983	**40.7**
**TN**	0.0239	0.0996	46	Spherical	0.985	**23.9**
**TP**	0.0321	0.0793	27	Spherical	0.981	**40.4**
**SOM**	8.05	29.59	19	Exponential	0.975	**27.2**
**TK**	1,25	3.55	4	Exponential	0.874	**35.1**
**TC**	0.02	0.07	18	Exponential	0.974	**28.5**

Based on Table 3, the coefficient of determination of the best fit model for soil nutrient indicators is high, indicating that the fitted model can well reflect the spatial characteristics of soil nutrients. The best fitting models of PH, TN, and TP were spherical models, while the best fitting models of SOM, TN, and TK were exponential models. The nugget effects of soil nutrient indicators in the study area were positive, indicating potential sampling or testing errors. The nugget value/base value of each nutrient index followed the order pH > TP > TK > TC > SOM > TN, and the nugget value/base values of pH, TP, TK, TC, and SOM were 40.7, 40.4, 35.1, 28.5, and 27.2%, showing moderate spatial autocorrelation. The nugget value/base value of pH, TP, TK, TC, and SOM range from 25% to 75%, accounting for a large proportion of the moderate spatial variability caused by both random factors and structural factors such as topography, fertilization, local ecological restoration measures or management level. The long-term application of large amounts of fertilizer resulted in a relatively stable range of soil acidity and alkalinity. The nugget value/base value of TN was 23.9%, which shows a strong spatial autocorrelation, indicating that the random factor contributes less to the spatial distribution of TN, and its spatial variation mainly depends on non-human structural factors such as soil parent material, soil type and natural mutation. The spatial dependence of soil SOM was moderate, while the spatial dependence of soil TN was strong. The result showed that the fertilization mode in this study area is mainly organic fertilizer, with less nitrogen fertilizer. Soil TP has less spatial dependence relative to the rest of the soil nutrients may related to the influence of unpredictable external factors such as frequent soil tillage, continuous crop harvesting and runoff.

The range reflects the size of the autocorrelation range of the variable space, which related to the observation scale and the interaction of various ecological processes affecting soil nutrients at the sampling scale. If the distances of observed values are within this range, the variables are spatially autocorrelated. Therefore, the range provides a measure to study the similarity range of a certain attribute. In the study area, the spatial autocorrelation range of soil nutrients differed, with values varying from 4 to 46 Km, indicating that the ecological processes affecting these soil factors play a role at different scales. The TN range in the soil surface of the study area was the largest (46 Km) and the ranges of pH, TP, SOM, TK and TC were 4 to 24 Km, following the order TP > pH > SOM > TC > TK. TN gradually changes with respect to other nutrient indicators in the soil. The spatial range of soil nutrients was all greater than the sampling interval (2.5 Km), indicating that the sampling strategy (the sampling scale) was sufficient for the study of the spatial distribution and pattern of soil properties.

### Spatial patterns of soil nutrients and pH

**Fig 4:**
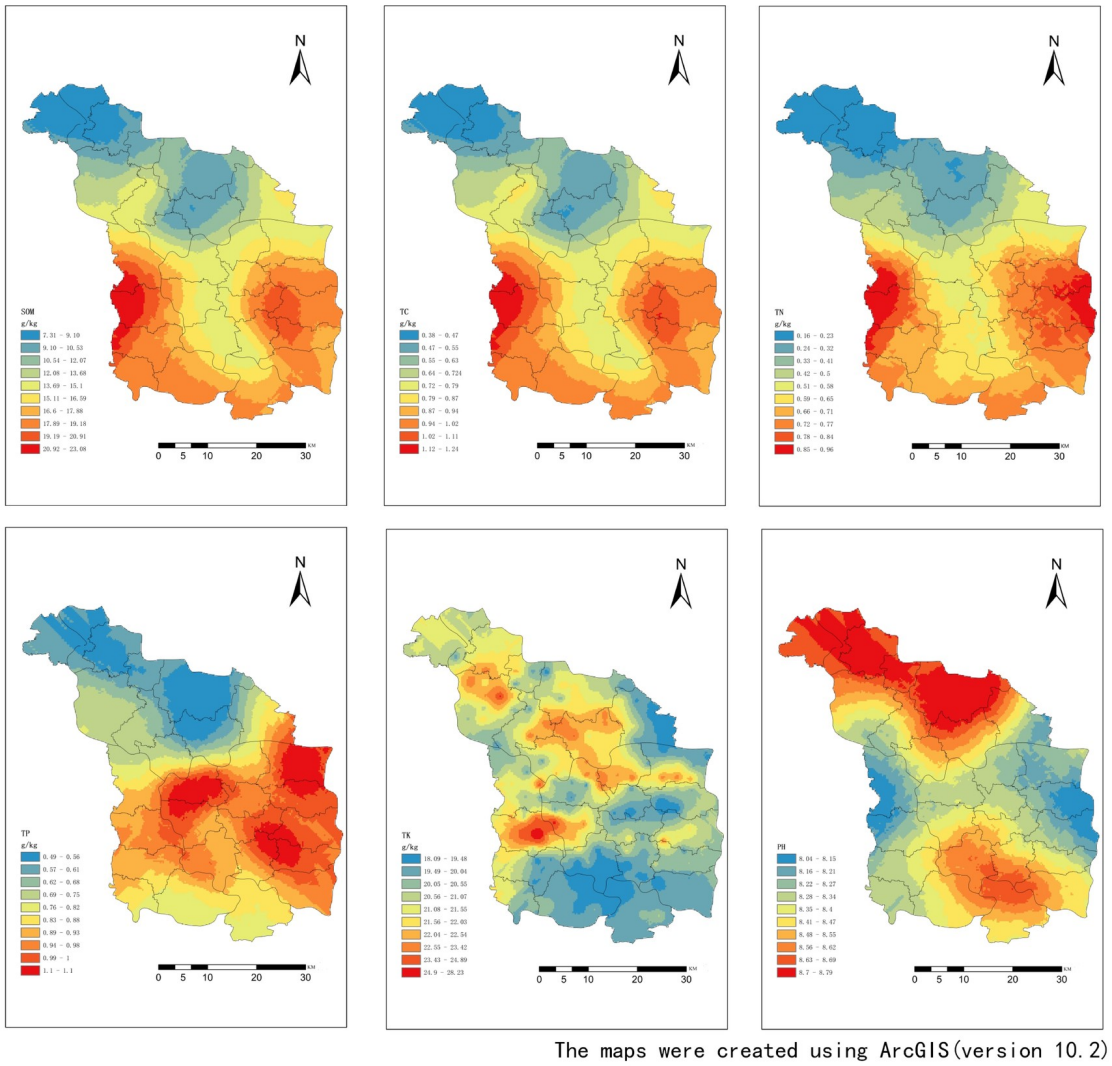
Spatial distribution of soil nutrients in the study area.

Soil has a complex morphology and evolution process, which affected by soil formation factors such as soil parent material, topography, climate, vegetation, and human disturbance activities, with a high spatial variability. In this study, we investigated the spatial distribution of soil nutrients and pH by generating the spatial equivalence map using kriging interpolation based on the semi-variograms. The spatial distribution of soil nutrients can reveal the evolution process of soil nutrients, and understand the relationship between soil and plants, the spatial pattern of vegetation and various ecological processes. The spatial distribution of soil TK in the study area can be expressed as a “plaque distribution” in Fig 4. Soil pH ranged between 8.04 and 8.79, indicating a weakly alkaline soil. The highest value was observed in the northern part of the study area, while the lowest values were found in the eastern and western parts. TN showed the following spatial distribution pattern: a gradual increase from north to south, with lowest values in the north; in the eastern and western regions, single peaks were observed. The parameters TP and TK showed a patchy and plaque gradient change; for TK, numerous plaques were observed. The spatial distribution characteristics were similar for SOM and TC, with scattered patches in the north and in the west, with a singe peak in the east of the study area. Such changes may aggravate the fragility of the ecosystem and lead to accelerated ecosystem degradation.

## Discussion

The content of soil organic matter plays a very important role in the evaluation of cultivated land quality [36]. The results of the study show that the content of TK and TP reached the second high level standard, the SOM content was at the middle to low level of the fourth grade, and the TN content was at the low level of the fifth grade. As a basic index of soil quality, soil SOM content is at a lower level, which may be due to the high rate of organic matter decomposition resulting from the complete removal of crop residues, continuous tillage and erosion of topsoil. The soil nutrient contents are influenced by human activities (such as reclamation, digging and farming), soil factors (such as soil type, soil parent material and topography) and climate factors (temperature and precipitation) [37]. Climate factors mainly affect the activity of microorganisms in soil, the leaching and migration process of nutrients and the productivity of vegetation itself, so as to affect the decomposition and input of nutrients in soil and further affect their content [38]. For organic matter, low temperature environment is conducive to the accumulation of organic matter in soil by inhibiting the decomposition of microorganisms, while large precipitation will increase the leaching of soil nutrients, thereby affecting the content of soil surface nutrients [39]. Previous researches found out that the contents of soil soluble organic carbon, monosaccharide, mineralized nitrogen, active inorganic phosphorus and organic phosphorus increased with the increase of drought intensity, while the contents of soil microbial biomass carbon, nitrogen, phosphorus and dissolved organic nitrogen decreased with the increase of drought intensity [40]. The direct influence of human activities on natural factors and soil nutrient content is also increasing. Xiao et al. (2017) [41] studied the effect of human interference on soil nutrient content, and the results showed that the extent of soil nutrient content affected by human activities gradually decreased with the increase of soil depth, while human activities such as grazing and reclamation significantly reduced soil surface nutrient content. At the same time, fertilizer content also directly affects the content of soil nutrients, which makes the spatial variation of soil nutrients produce bigger change [42]. The degree of interference of human activities on soil nutrient distribution cannot be ignored and should take this factor into consideration in the future analysis, which establishes a more scientific and perfect the index system. To improve this situation, it is necessary to apply appropriate amount of bio-organic fertilizer during farming to promote a virtuous cycle of soil fertility [43]. In addition, policymakers should continue to research and promote various technical models that promote the healthy development of farmland, such as straw crushing and green manure planting. Existing studies have shown that there is a very significant correlation between alkali- hydrolyzed nitrogen and organic matter content [44]. We analyzed the correlation analysis of soil nutrients (Table 4). There is a very significant positive correlation between the TN and organic matter of the cultivated soil in the study area with the correlation coefficient of the two is 0.841 (Table 4). Previous studies have also reported that soil organic matter affects the storage and transformation of nitrogen [45] and even releases alkali- hydrolyzed nitrogen [46]. The relationship between organic matter and TN is close, which can explain the distribution pattern of TN content in the study area on a spatial scale is similar to organic matter. The correlation between TN content and aftercrop yield and nitrogen uptake is more significant [47]. The main crops in the study area are wheat, corn, peanut, sweet potato, which are nitrogen-loving and phosphorus-loving crops. Alkali-hydrolyzed nitrogen is absorbed and utilized by these crops and sampling time is mostly during crop harvesting, which lead to a lower level of alkali- hydrolyzed nitrogen in the study area [48]. In addition, the content of alkali- hydrolyzed nitrogen and available phosphorus plays a significant role in characterizing soil fertility [49]. The total nitrogen content of the soil in the study area is generally low, and the content of alkali-hydrolyzed nitrogen is relatively low. Deep application of chemical fertilizers can improve this problem. In the study area, the content of TP is relatively high, but the spatial distribution is uneven. The reason for this difference may be related to the amount of phosphate fertilizer applied during the planting process. Less rainfall makes TK not easy to be lost, and ultimately leads to higher TK content in the study area. The pH value is negatively correlated with the values of SOM, TN, TP and TC (Table 4), indicating that with the increase of soil alkalinity, the soil SOM, TN, TP and TC decreased. Therefore, it is necessary to maintain a reasonable amount of nitrogen fertilizer, improve the efficiency of nitrogen fertilizer use and fertilization management level. Excessive application of nitrogen fertilizer will not only destroy the balance of soil carbon and nitrogen coupling, but also aggravate agricultural pollution problems such as soil acidification and compaction [50]. In addition, local policymakers should be promote the cultivation of green manure and straw application, encourage to apply more organic fertilizers, improve soil physical and chemical properties and soil carbon and nitrogen content, and achieving increased food production and sustainable agricultural development.

**Table 4:** Correlation analysis of soil nutrients.

	**PH**	**TN**	**TP**	**SOM**	**TK**	**C**
**PH**	1	-.608**	-.438**	-.535**	-0.01	-.531**
**TN**		1	.575**	.841**	-.154*	.836**
**TP**			1	.560**	-.165*	.558**
**SOM**				1	-.131*	.993**
**TK**					1	-0.113
**C**						1

** Significantly correlated at the 0.01 level (two-sided).

* Significantly correlated at the 0.05 level (two-sided).

We observed a spatial variability of TN, TP, TK, TC, and SOM throughout the study area. The coefficient of determination of the best fit model of the soil nutrient index was relatively high, indicating that the fitted model can well reflect the spatial characteristics of the soil nutrients. The best fitting models of pH, TN, and TP were spherical models, while those of SOM, TN, and TK were exponential models. The nugget effects of soil nutrient indicators in the study area were positive, indicating sampling and testing errors. Soil pH, TP, TK, TC, and SOM had the moderate spatial variability, which was caused by both random factors and structural factors such as topography, fertilization, local ecological restoration measures or management level. Soil TN showed a strong spatial correlation, mainly due to parent land topography and soil type. Therefore, if the spatial heterogeneity of each soil factor is ignored, the recommended fertilization amount is still given based on the average value of soil nutrients, which will not only affect crop production efficiency and fertilizer utilization, but may also cause greater environmental pollution. May cause greater pollution of the environment. Therefore, on the basis of the above research, according to the given variation function model, use geostatistics software to perform Kriging spatial interpolation mapping, especially for soil TN, TP spatial interpolation mapping, which is helpful to identify high nitrogen and phosphorus nutrients. The identification of areas and the implementation of soil nutrient zoning management are of great significance to reducing the loss of nitrogen and phosphorus nutrients and the treatment of agricultural non-point source pollution in the Guo River Basin. The spatial range of soil nutrients in this study area is greater than the sampling interval. The range is mainly related to the size of the study area and the sampling intensity. The larger of the range indicates the stronger of the soil heterogeneity at the sampling scale [1]. For example, Behera and Shukla (2015) [51] measured different range ranges (0.044–11.936 Km) in acidic soils in India, which was due to comprehensive influence of different parent soil climatic conditions and land management practices. Ozgoz et al. (2013) [52] found that soil quality can be affected through farm soil management measures such as tillage fertilization and water management and crop rotation. Overall, if the spatial variability of soil factors is neglected, the fertilizer amount cannot be adapted, which not only affects crop production efficiency and fertilizer use, but also causes considerable environmental pollution [53].

**Fig 5:**
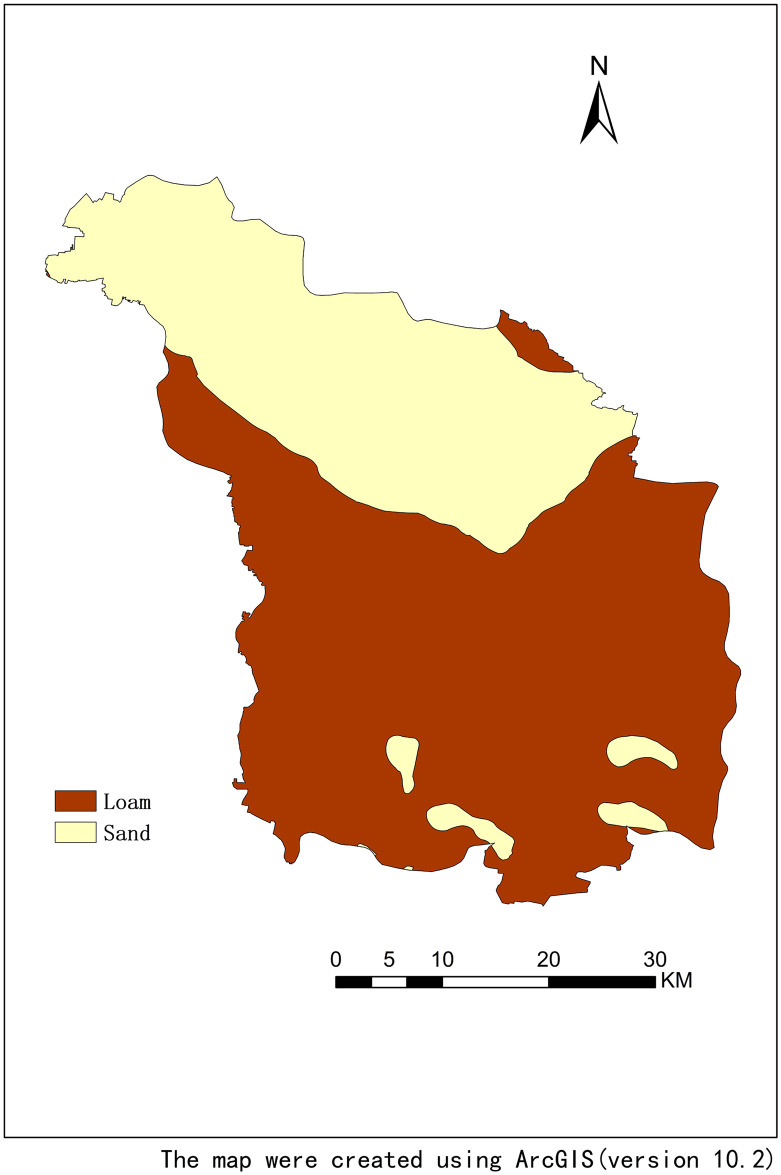
Distribution map of soil texture.

Using the geostatistical module of ArcGIS 10.2 to make a 3D distribution map of each nutrient content and geographic location can intuitively understand the large-scale spatial distribution trend of each nutrient. It is convenient to study the spatial variability at small scale if here is a reasonable distribution trend and eliminate the large spatial distribution trend. In Fig 3, the trend line of each soil nutrient passed through the projected area of the sample point in the east-west and south-north directions, suggesting the overall variation trend of each nutrient. Soil SOM, TC, TN and TP all show an increasing trend along the north-south direction, with low content in the north and high content in the south, while TK showed the opposite trend. Soil pH shows a trend of low in the middle and high in the north and south. The variation trend of soil nutrient is affected by both structural factors and random factors. In addition to the factors such as slope, aspect, altitude, curvature, different soil types and soil textures can greatly affect the content of soil nutrients. Table 5 showed the soil nutrient content of different soil types. We can found that the nutrient content of the fluvo-aquic soil and the alkalized fluvo-aquic soil are higher than that of the meadow soil. The alkalized fluvo-aquic soil has the highest organic matter content, and the meadow terroir has the lowest organic matter content, while the content of soil TK is the highest. Table 6 analyzed the soil nutrient content of different soil texture. In Table 6, SOM, TN and TP all show the characteristics that the more viscous of soil texture, the higher of the average nutrient content. The results were consistent with the study of Zhang et al (2014) [54]. We also draw the distribution map of soil texture as showed in Fig 5. The northern part of the study area is sandy soil, and the southern part is mainly loam with spotted sandy soil. It can be seen that the cultivated land in the southern part of the study area is the most sticky, while the northern part is relatively sandy. Overall, the spatial distribution of soil nutrients is in well consistent with the spatial distribution of soil texture.

**Table 5:** Soil nutrient content of different soil types.

			TN	TP	SOM	TK	TC
**Soil type**	Subclass	N	Content(g/kg)	Content(g/kg)	Content(g/kg)	Content(g/kg)	Content(g/kg)
**Fluvo-aquic soils**	Fluvo-aquic soil	222	0.55±0.26	0.84±0.26	15.00±4.60	20.85±1.84	0.79±0.24
**Aeolian sandy soils**	Alkaline alluvial soil	10	0.48±0.24	0.67±0.23	15.95±6.13	20.87±1.39	0.84±0.32
	Meadow Terroir	7	0.30±0.13	0.63±0.29	9.23±2.33	22.49±1.35	0.49±0.12

**Table 6:** Soil nutrient content of different soil texture.

		TN	TP	SOM	TK	TC
**Soil texture**	N	Content(g/kg)	Content(g/kg)	Content(g/kg)	Content(g/kg)	Content(g/kg)
**sand**	92	0.37±0.21	0.65±0.22	11.86±4.03	21.44±1.51	0.62±0.21
**loam**	147	0.65±0.23	0.93±0.23	16.71±4.11	20.56±1.92	0.88±0.21

## Conclusion

Determining the distribution of soil nutrients is the first step to reduce agricultural non-point source pollution, reduce agricultural production costs, increase agricultural production and realize precision agriculture. Using Kriging interpolation to generate soil nutrient distribution maps is an effective method to determine the spatial distribution of soil nutrients.

1. The descriptive statistics of each sample point in the study area showed that the average content of SOM, TC, TN, TP, TK, and PH is 14.85g/kg, 0.78/kg, 0.54/kg, 0.83/kg, 20.90/kg and 8.43/kg respectively. The content of TK and TP is high, but SOM and TN are low. The coefficient of variation of TK and PH is small, showing a weak degree of variation. The coefficient of variation of SOM, TC, TN and TP is of medium degree of variation. Among them, the coefficient of variation of TN is larger, which is related to the chemical behavior of N in the soil and the current status of phosphate fertilizer application. Because of the low utilization rate of N applied to the soil, N residue caused uneven distribution of N in the soil.

2. Soil nutrient heterogeneity is the result of the combined effect of structural factors and random factors. TN is mainly affected by structural factors and shows strong spatial correlation. The SOM, TC, TP, TK and PH shows medium spatial correlation and mainly affected by both structural and random factors. The spatial distribution of soil nutrients is highly consistent with the spatial distribution of soil types and textures. Therefore, suitable soil erosion control measures should be adopted according to the different soil types and textures characteristics in the River Basin region for soil fertility recovery.

In addition, to improve our understanding of the spatial variability of SOM, TC, TN, TP, TK, and pH in response to ecological restoration, the long-term spatiotemporal dynamics of SOM, TC, TN, TP, TK, and pH should be examined in future studies.

## Abbreviations

BP: Back Propagation
CO_2_: Carbon dioxide
CV: Coefficient of variation
DEM: Digital Elevation Model
pH: Potential of Hydrogen
R^2^: Coefficient of determination
RBF: Radial Basis Function Neural Network
RSS: Residual Sum of Squares
SAS: Statistical Analysis System
SOM: Soil organic matter
SPSS: Statistical Program for Social Sciences
TC: Total carbo
TK: Total potassium
TN: Total nitrogen
TP: Total phosphorous
UTM: Universal Transverse Mercator
